# Organisational determinants and consequences of diagnostic discrepancy in two large patient groups in the emergency departments: a national study of consecutive episodes between 2008 and 2016

**DOI:** 10.1186/s12873-021-00538-9

**Published:** 2021-11-22

**Authors:** Line Stjernholm Tipsmark, Børge Obel, Tommy Andersson, Rikke Søgaard

**Affiliations:** 1 DEFACTUM, Central Denmark Region, Olof Palmes Allé 15, 8200 Aarhus N, Denmark; 2 Department of Public Health, Aarhus University, Bartholins Allé 2, 8000 Aarhus C, Denmark; 3 DESIGN EM – Research Network for Organizational Design and Emergency Medicine, Fuglesangs Allé 4, 8210 Aarhus V, Denmark; 4 Department of Management, Aarhus University, Fuglesangs Allé 4, 8210 Aarhus V, Denmark; 5 Interdisciplinary Centre for Organizational Architecture, Aarhus University, Fuglesangs Allé 4, 8210 Aarhus V, Denmark; 6 Regional Hospital West Jutland, Gl. Landevej 61, 7400 Herning, Denmark; 7 Department of Clinical Research, University of Southern Denmark, J.B. Winsløws Vej 4, 5000 Odense C, Denmark

**Keywords:** Emergency service, hospital, Denmark, Diagnostic error, Organization and administration, Emergency medicine

## Abstract

**Background:**

Diagnostic discrepancy (DD) is a common phenomenon in healthcare, but little is known about its organisational determinants and consequences. Thus, the aim of the study was to evaluate this among selected emergency department (ED) patients.

**Method:**

We conducted an observational study including all consecutive ED patients (hip fracture or erysipelas) in the Danish healthcare sector admitted between 2008 and 2016. DD was defined as a discrepancy between discharge and admission diagnoses. Episode and department statistics were retrieved from Danish registers. We conducted a survey among all 21 Danish EDs to gather information about organisational determinants. To estimate the results while adjusting for episode- and department-level heterogeneity, we used mixed effect models of ED organisational determinants and 30-day readmission, 30-day mortality and episode costs (2018-DKK) of DDs.

**Results:**

DD was observed in 2308 (3.3%) of 69,928 hip fracture episodes and 3206 (8.5%) of 37,558 erysipelas episodes. The main organisational determinant of DD was senior physicians (nonspecific medical specialty) being employed at the ED (hip fracture: odds ratio (OR) 2.74, 95% confidence interval (CI) 2.15–3.51; erysipelas: OR 3.29, 95% CI 2.65–4.07). However, 24-h presence of senior physicians (nonspecific medical specialty) (hip fracture) and availability of external senior physicians (specific medical specialty) (both groups) were negatively associated with DD. DD was associated with increased 30-day readmission (hip fracture, mean 9.45% vs 13.76%, OR 1.46, 95% CI 1.28–1.66, *p* < 0.001) and episode costs (hip fracture, 61,681 DKK vs 109,860 DKK, log cost 0.58, 95% CI 0.53–0.63, *p* < 0.001; erysipelas, mean 20,818 DKK vs 56,329 DKK, log cost 0.97, 95% CI 0.92–1.02, *p* < 0.001) compared with episodes without DD.

**Conclusion:**

DD was found to have a negative impact on two out of three study outcomes, and particular organisational characteristics seem to be associated with DD. Yet, the complexity of organisations and settings warrant further studies into these associations.

## Background

Diagnostic error is a common phenomenon in healthcare, especially in patients hospitalised via the emergency departments (EDs) [1]. ED patients presents with diagnostic error rates from 0.6–64% [2–4]. Some of this variation may be rooted in differences in how diagnostic error is defined, viz. as primary missed diagnosis, unintentionally delayed diagnosis, wrong diagnosis and diagnostic discrepancy (DD) [2, 5–8].

Diagnostic error is associated with both cognitive and system-related factors. Cognitive factors include inadequate data synthesis. Among system-related factors, organisational issues were the primary source of diagnostic error [5]. Some of these issues may be related to the ED environment, which is known to be unpredictable and stressful. Moreover, diagnostic decision-making is complex, especially in the ED due to an overly broad patient spectrum compared to other medical specialties. Overall, this seems to increase the risk of incorrect admission diagnosis [1, 9, 10], and poor diagnostic quality could potentially impair patient safety [6].

The diagnostic error literature primarily contains single- or multi-centre studies [4, 6, 7]. Thus, national studies are lacking. Diagnostic error is reviewer dependent and commonly detected by review of medical records, which requires significant resources [4–7, 11]. To provide larger studies, another measure of diagnostic error is thus needed. DD is defined as a discrepancy between discharge and admission diagnoses and DD is a precondition for diagnostic error [6]. DD is an objective measure which can be obtained from register data, making larger analyses possible. Furthermore, previous studies have primarily included patients with diagnostic errors to determine the causes of DD and little is known about organisational determinants and consequences of DD [6]. Thus, our aim was to analyse the organisational determinants and effects of DD at a national level of ED episodes between 2008 and 2016.

## Method

### Study design and setting

The study was designed as an observational study of emergency episodes at all 21 Danish EDs. All in- and outpatient emergency episodes treated at somatic hospitals in Denmark from 1 January 2008 to 10 September 2016 (most recent register data available) were included and followed up to 30 days after discharge. Episodes were included if the patient was ≥18 years and discharged with an International Classification of Diseases (ICD) version 10 (ICD-10) code of hip fracture (DS720, DS721, DS721A, DS721B, DS722) or erysipelas (DA469). These diagnoses were chosen because they account for a high ED volume and featured a stable diagnostic and treatment history throughout the study period. DD was identified in the included population. Due to the study design, patients could be registered with more than one episode during the study period. Most EDs have a catchment area of 100,000–400,000 citizens. The small EDs have restricted access to specialised equipment and senior physician counselling, whereas the larger EDs generally have the required in-house resources [12, 13]. However, almost half of the EDs (43%) do not have 24-h senior physician coverage [13]; and senior physician coverage seems to be associated with hospital size and political decision-making at the regional level.

### Diagnostic discrepancy

DD was defined as discrepancies between discharge and admission diagnoses. We classified DD according to a previously used classification [6] (Table 1) into ‘identical’ diagnoses: discharge and admission diagnoses were the same; ‘more precise’ diagnoses: the discharge diagnosis was more precise than the admission diagnosis but in the same diagnostic category; ‘hierarchically different’ diagnoses: the discharge diagnosis was listed among the secondary admission diagnoses; and ‘diagnostically different’ diagnoses: the discharge diagnosis was not among the admission diagnoses. Examples can be found in Table 1. We dichotomised DD into two definitions; Thus, definition 1 comprised ‘hierarchically’ and ‘diagnostically different’ DDs; definition 2 comprised only ‘diagnostically different’ DDs.

**Table 1:** Definition of diagnostic discrepancy [6]

Outcome	Discharge diagnosis compared with admission diagnosis	Explanation	Example
No diagnostic discrepancy	Identical	The discharge diagnosis was the same as the admission diagnosis	
	More precise	The discharge diagnosis was more precise than the admission diagnosis	A patient is admitted with S70.0 Fracture of femur and is discharge with S72.2 Subtrochanteric fracture
Diagnostic discrepancy	Hierarchically different	The discharge diagnosis was listed as a secondary admission diagnosis	A patient is discharged with erysipelas, which was a secondary diagnosis at admission
	Diagnostically different	The discharge diagnosis was not among the admission diagnoses. The definition is given if none of the previous descriptions match the episode	A patient is admitted with dehydration as admission diagnosis and discharged with hip fracture

### Variables and data sources

The organisational determinants under investigation were senior physicians employed at the ED (nonspecific medical specialty), presence of senior physicians 24-h a day (nonspecific medical specialty), availability of external senior physicians (specific medical specialty), whether the EDs used flow coordinators and multidisciplinary teams, if the ED had decision authority (the authority to make treatment decisions without consulting physicians from other departments) and ED facilities located in a single building. These organisational determinants are key when defining the ED organisational design. Moreover, information processing, and hence information gathering for making a diagnoses, depends upon the organisational design and these parameters [12, 14–17]. The outcomes under investigation were 30-day readmission defined as acute readmission to any hospital department within 30 days after discharge excluding accidents, mental disease and cancer treatment [18]; 30-day mortality defined as death within 30 days after the diagnosis was given [19]; and episode costs defined as resource use from admission to discharge. Episode costs were stated in DKK 2018 and log transformed. Episode characteristics included gender, age and comorbidity based on the Elixhauser Comorbidity Index [20–22]*;* and department characteristics included annual episode volume, teaching status, means of 30-day readmission, 30-day mortality and episode costs. Department characteristics were based on episode level means during the year preceding the episodes at the admission hospital*.*

Data to construct all patient and department characteristics were retrieved from the Danish National Patient Register [23], Central Person Registry [24] and the Reference Cost Database [25]. Data on organisational determinants and implementation time were retrieved from a survey completed in 2017 by all 21 Danish EDs. The Reference Cost database did not contain 2016 data, and it was the only database with missing data in our sample (11%, excluding 2016 data). Missing department costs were imputed with data from the year before (last valued carried forward) to keep the episodes from the affected department in the multilevel analyses [26, 27]. We did not expect missing costs to be associated with an observed or unobserved variable related to the outcome (we compared baseline episode and department characteristics for the episodes with and without missing costs) [28]. Hence, missing cost data were assumed to be missing completely at random (MCAR). Mixed effect models used in this study are suitable for handling missing data [29, 30].

### Statistical tests

To compare episode and department characteristics with and without DD, summary statistics of binary variables were tested by the Pearson chi-square test and continuous variables were tested by the Wilcoxon rank-sum (Mann-Whitney) test, and the significance level was set at *p* < 0.05.

### Mixed effect models

Organisational determinants and effects of DD were analysed in mixed effects models while adjusting for episode and department heterogeneity. The mixed effects models rely on hierarchical data at episode and department level to handle the intra-unit correlation that occurs where cluster-level intervention is analysed at the individual patient or episode level [31, 32]. Furthermore, time (year) was included in mixed effects models to account for secular trends. In the descriptive analyses, DD definition 1 was applied; and in the mixed effects models, the results of both definition 1 and 2 were applied. Due to a small number of clusters (21 EDs), we applied small sample correction to construct confidence intervals [33, 34].

## Results

**Fig. 1:**
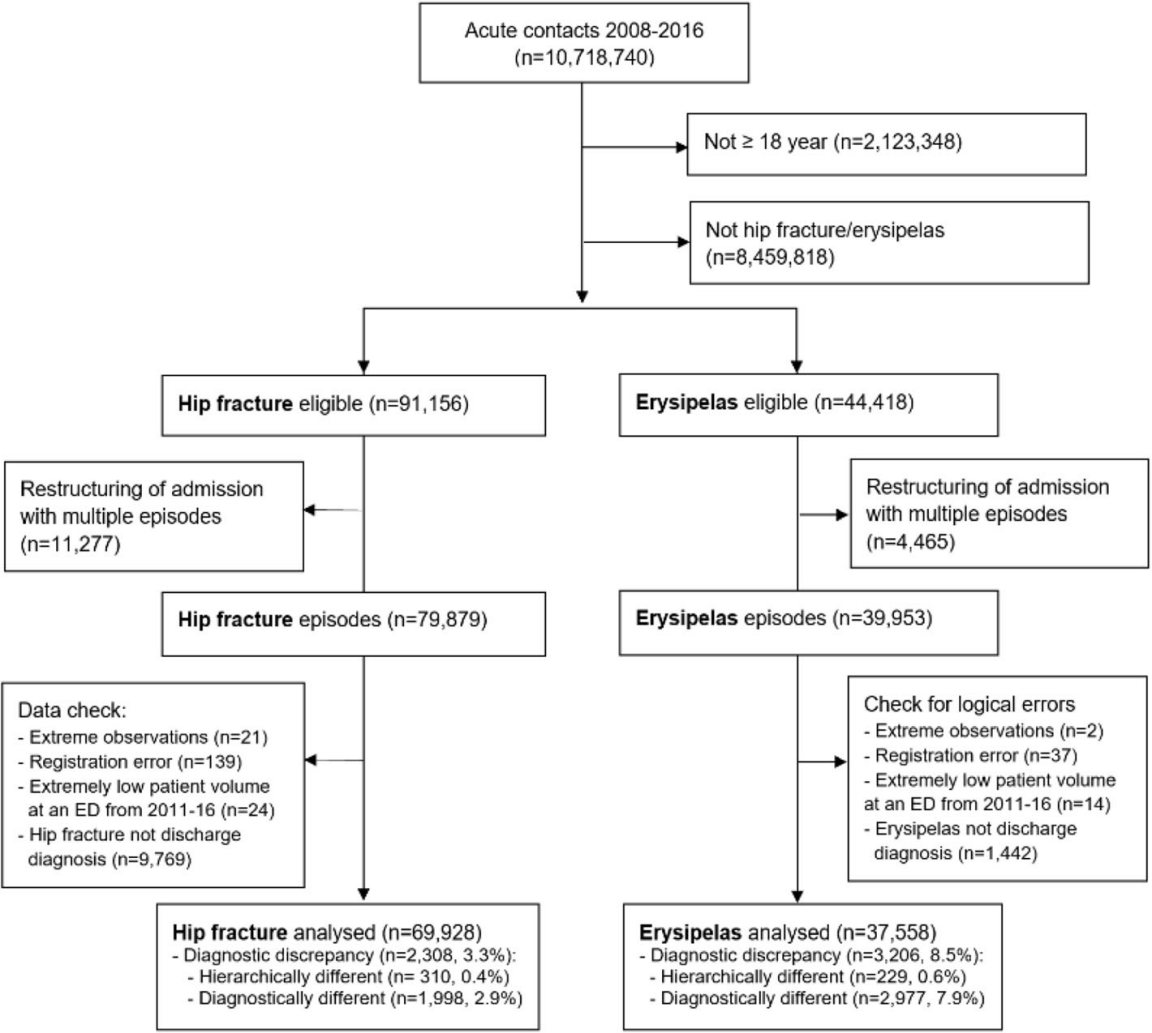
Flow diagram

**Fig. 2:**
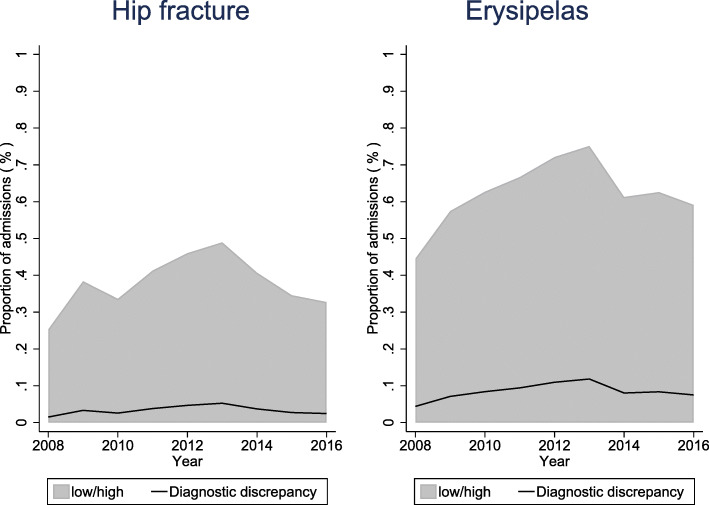
Proportion of diagnostic discrepancy over time. Grey area, 95% confidence interval

In the 9-year study period, 69,928 episodes were registered with hip fracture as a discharge diagnosis and 37,558 episodes were registered with erysipelas as a discharge diagnosis. DD was detected in 2308 (3.3%) hip fracture episodes and 3206 (8.5%) erysipelas episodes (Fig. 1). The proportion of DD was almost constant during the study period; yet, a small peak was observed around 2013 (Fig. 2).

Hip fracture episodes with DD were characterised by greater complexity as evidenced in an almost two-fold increased comorbidity index score (0.27 vs 0.50, *p* < 0.001), a higher risk of 30-day readmission (0.09 vs. 0.14, *p* < 0.001) and substantially increased episode costs (61,682 DKK vs. 109,860 DKK, *p* < 0.001) (Table 2). For erysipelas episodes, the same pattern was observed for comorbidity (0.36 vs. 0.69, *p* < 0.001) and episode costs 20,818 (DKK vs. 44,645 DKK, *p* < 0.001), whereas 30-day readmission was similar (0.13 vs. 0.14, *p* = 0.283) and 30-mortality was higher (0.01 vs. 0.02, *p* < 0.001). In terms of department characteristics, hip fracture DD were more often observed at teaching hospitals and at EDs with a lower hip fracture volume and increased ED costs. The same pattern was observed for department characteristics of erysipelas episodes with and without DD.

**Table 2:** Episode and department characteristics for consecutive ED patients between 2008 and 2016

	Hip fracture (***n*** = 69,928)			Erysipelas (***n*** = 37,558)		
	No diagnostic discrepancy *n* = 67,620 (96.7%)	Diagnostic discrepancy^1^ *n* = 2308 (3.3%)	*p* value^2^	No diagnostic discrepancy *n* = 34,352 (91.5%)	Diagnostic discrepancy^1^ *n* = 3206 (8.5%)	*p* value
**Episode**						
Male gender (%)	0.31 (0.46)	0.38 (0.49)	< 0.001	0.57 (0.49)	0.56 (0.50)	0.093
Age (years)	78.73 (12.30)	77.89 (12.63)	< 0.001	61.46 (17.82)	67.55 (16.33)	< 0.001
Elixhauser Index^3^	0.27 (0.65)	0.50 (0.88)	< 0.001	0.36 (0.74)	0.69 (0.96)	< 0.001
30-day readmission (%)	0.09 (0.29)	0.14 (0.35)	< 0.001	0.13 (0.34)	0.14 (0.34)	0.283
30-day mortality (%)	0.09 (0.29)	0.10 (0.30)	0.139	0.01 (0.11)	0.02 (0.15)	< 0.001
Episode cost (2018-DKK)	61,682 (45,458)	101,823 (78,770)	< 0.001	20,818 (27,610)	44,645 (44,191)	< 0.001
**Departments**						
Teaching status (%)	0.22 (0.41)	0.32 (0.47)	< 0.001	0.17 (0.38)	0.37 (0.48)	< 0.001
Episode volume (n)	549 (216)	502 (189)	< 0.001	350 (252)	273 (166)	< 0.001
30-day readmission (%)	0.10 (0.05)	0.10 (0.03)	< 0.001	0.13 (0.03)	0.13 (0.03)	< 0.001
30-day mortality (%)	0.10 (0.02)	0.10 (0.02)	0.360	0.02 (0.01)	0.02 (0.01)	< 0.001
Episode cost (2018-DKK)	73,048 (22,766)	80,913 (24,538)	< 0.001	32,047 (22,362)	33,563 (21,300)	< 0.001

*ED* Emergency department,

^1^Hierarchically and diagnostically different diagnoses were defined as diagnostic discrepancy

^2^Binary variables were tested by the Pearson chi-square test and continuous variables were tested by the Wilcoxon rank-sum test, and the significance level was set at *p* < 0.05

^3^ Total, unweighted score (the 19 individual variables cannot be shown according to the General Data Protection act)

Variables are reported as episode and department means (standard deviation)

Observation for suspected disease or condition, unspecified (Z03.9) was the most frequent admission diagnosis (hip fracture 14.69% and erysipelas 24.45%) among DD episodes (Table 3).

**Table 3:** The 10 most frequent admission diagnoses among patients with diagnostic discrepancy

Discharge diagnosis	ICD-10 code	Admission diagnosis	Frequency (%)
**Hip fracture**	Z03.9	Observation for suspected disease or condition, unspecified	339 (14.69)
	S70.0	Contusion of hip	190 (8.23)
	Z03.8	Observation for other suspected diseases and conditions	120 (5.20)
	Z47.8	Other specified orthopaedic follow-up care	79 (3.42)
	Z04.9	Examination and observation for unspecified reason	60 (2.69)
	S32.5	Fracture of pubis	43 (1.86)
	J18.9	Pneumonia, unspecified	41 (1.78)
	R52.9	Pain, unspecified	39 (1.69)
	E86.9	Dehydration	38 (1.65)
**Erysipelas**	Z03.9	Observation for suspected disease or condition, unspecified	784 (24.45)
	Z03.8	Observation for other suspected diseases and conditions	245 (7.64)
	A41.9	Sepsis, unspecified	154 (4.80)
	A26.9	Erysipeloid, unspecified	109 (3.40)
	R50.9	Fever, unspecified	107 (3.34)
	Z04.9	Examination and observation for unspecified reason	79 (2.46)
	A49.9	Bacterial infection, unspecified	79 (2.46)
	E86.9	Dehydration	73 (2.28)
	J18.9	Pneumonia, unspecified	67 (2.09)
	M76.9	Enthesopathy of lower limb, unspecified	50 (1.56)

*ICD-10* International Classification of Diseases, version 10

### Determining diagnostic discrepancy by emergency department organisational characteristics

Using DD definition 1, we found hip fracture episodes to be associated with senior physician employment (OR 2.75, 95% CI 2.15–3.50), multidisciplinary teams (OR 1.50, 95% CI 1.19–1.88) and decision authority (OR 1.83 95% CI 1.47–2.27) (Table 4). Inversely, availability of external senior physicians (OR 0.50, 95% CI 0.39–0.65), facilities in one building (OR 0.65, 95% CI 0.52–0.81) and presence of senior physicians 24 h a day (OR 0.68, 95% CI 0.53–0.88) were negatively associated with DD. For erysipelas episodes, DD was associated with senior physician employment (OR 3.29, 95% CI 2.65–4.08), decision authority (OR 1.80, 95% CI 1.49–2.18), multidisciplinary teams (OR 1.40, 95% CI 1.15–1.70) and facilities in one building (OR 1.39, 95% CI 1.13–1.73). External senior physician (OR 0.44, 95% CI 0.36–0.54) and flow coordinator (OR 0.69, 95% CI 0.55–0.84) were negatively associated with DD.

**Table 4:** Organisational determinants of diagnostic discrepancy for consecutive ED patients between 2008 and 2016

Organisational determinants	Hip fracture (***n*** = 69,928)		Erysipelas (***n*** = 37,558)	
	Definition 1^a^ OR (95% CI)	Definition 2^b^ OR (95% CI)	Definition 1^a^ OR (95% CI)	Definition 2^b^ OR (95% CI)
Senior physicians employed at the ED	2.75 (2.15–3.50)	3.59 (2.72–4.74)	3.29 (2.65–4.08)	3.59 (2.86–4.50)
Senior physicians 24-h a day	0.68 (0.53–0.88)	0.64 (0.47–0.84)	1.09 (0.86–1.37)	1.23 (0.96–1.56)
External senior physicians	0.50 (0.39–0.65)	0.50 (0.38–0.66)	0.44 (0.36–0.54)	0.41 (0.33–0.50)
Flow coordinator	0.97 (0.75–1.23)	0.97 (0.75–1.28)	0.69 (0.55–0.84)	0.61 (0.49–0.75)
Multidisciplinary team	1.50 (1.19–1.88)	1.42 (1.10–1.82)	1.40 (1.15–1.70)	1.52 (1.24–1.85)
Decision authority	1.83 (1.47–2.27)	1.94 (1.52–2.47)	1.80 (1.49–2.18)	1.77 (1.45–2.15)
Facilities in one building	0.65 (0.52–0.81)	0.52 (0.41–0.67)	1.39 (1.13–1.73)	1.39 (1.11–1.75)

*OR* odds ratio, *CI* confidence interval

^a^Hierarchically and diagnostically different diagnoses were defined as diagnostic discrepancy; hip fracture *n* = 2308, erysipelas *n* = 3206

^b^Diagnostically different diagnoses were defined as diagnostic discrepancy; hip fracture *n* = 1998, erysipelas *n* = 2977

Results are coefficients from mixed effects models expressing the association between diagnostic discrepancy and the emergency department organisational characteristics. All estimates are adjusted for all covariates shown in Table 2 (episode-level age, gender and comorbidity and department-level teaching status, episode volume, and average 30-day readmission, 30-day mortality and episode costs)

The definition used for DD appeared to play a role primarily for senior physician employment across hip fracture episodes, with the largest OR for definition 2 (definition 1 OR 2.75, 95% CI 2.15–3.50; definition 2 OR 3.59 95% CI 2.72–4.74). Definition 2 only included the ‘diagnostically different’ diagnoses; therefore, the discharge diagnosis was not included at admission as is the case for definition 1. Hence, the probability of diagnostic error is assumed to be larger in definition 2.

### Consequences of diagnostic discrepancy

Using DD definition 1, we found that DD among hip fracture episode resulted in a 45% increased 30-day readmission rate (*p* < 0.001), which corresponds to an average increase in 30-day readmission from 9.45% for episodes without DD to 13.69% for episodes with DD (Table 5). Episode costs rose by 78% (*p* < 0.001), corresponding to an increase from an average of 61,682 DKK for episodes without DD to 109,860 DKK for episodes with DD. DD among erysipelas episode increased episode costs by 171% (*p* < 0.001), viz. an increase from 20,818 DKK for episodes without DD to 56,329 DKK for episodes with DD. Outcomes were quite similar among the two definitions, and did not affect the statistical significance of the outcome.

**Table 5:** Consequences of diagnostic discrepancy for consecutive ED patients between 2008 and 2016

Diagnostic discrepancy	Hip fracture (***n*** = 69,928)			Erysipelas (***n*** = 37,558)		
	30-day readmission OR (95% CI)	30-day mortality OR (95% CI)	Episode costs Log cost (95% CI)	30-day readmission OR (95% CI)	30-day mortality OR (95% CI)	Episode costs Log cost (95% CI)
Definition 1^a^	1.45 (1.27–1.65)	1.10 (0.94–1.29)	0.58 (0.53–0.63)	0.98 (0.87–1.10)	1.20 (0.91–1.57)	1.00 (0.93–1.05)
Definition 2^b^	1.41 (1.23–1.62)	1.07 (0.90–1.27)	0.57 (0.52–0.62)	1.00 (0.89–1.13)	1.23 (0.92–1.61)	0.98 (0.92–1.04)
**Model characteristics**						
Episode (n)	69,330	69,324	56,235	37,296	37,091	28,844
Department (n)	21	21	21	21	21	21
Min episodes per ED	330	330	238	110	110	110
Max episodes per ED	6868	6867	6367	5084	5036	3566
Wald chi2	1041*	3464*	12,520*	2752*	3474*	9161*

*OR* Odds ratio, *CI* Confidence interval

^a^Hierarchically and diagnostically different diagnoses were defined as diagnostic discrepancy; hip fracture *n* = 2308, erysipelas *n* = 3206

^b^Diagnostically different diagnoses were defined as diagnostic discrepancy; hip fracture *n* = 1998, erysipelas *n* = 2977

Results are coefficients from mixed effects models expressing the effect of diagnostic discrepancy on 30-day readmission, 30-day mortality and episode costs. All estimates are adjusted for all covariates shown in Table 2 (episode-level age, gender and comorbidity and department-level teaching status, episode volume, and average 30-day readmission, 30-day mortality and episode costs)

*The significance level was set at *P* < 0.001

## Discussion

In this nationwide study of consecutive emergency episodes with relatively common diagnoses, DD was observed in 3.3% of hip fracture episodes and 8.5% of erysipelas episodes. DD had direct consequences for episode outcomes. Thus, 30-day readmission was increased by 45% for hip fracture episodes, and episode costs were increased by 79% for hip fracture episodes and 171% for erysipelas episodes. Senior physician employment at the ED – as opposed to external senior physicians being on call – appeared to be the strongest determinant of DD followed by decision authority and multidisciplinary team.

Several studies have assessed mechanisms leading to suboptimal diagnoses [1, 3, 5–7, 9]. One of these studies assessed organisational factors [5], finding that diagnostic errors were associated with system-related and cognitive factors. The former covered teamwork, for example, as also found in the present study. A few studies have assessed the potential consequences of DD and mainly assessed outcome in terms of costs, which they found to be increased [35, 36]. We also identified a recent study assessing consequences of DD in terms of health (in-hospital mortality) and quality of care (length of stay) [6]. This study found both outcomes to be significantly increased among patients with DD. This study resembles our study in terms of methodology. Hence, both used the same definition of DD and both reported health and quality of care outcomes. However, we focused on 30-day outcome, whereas Hautz et al. [6] focused on outcomes during hospital stay only. In-hospital mortality was included in our 30-day measure, since it is recorded as from the day of diagnosis (hip fracture or erysipelas). The only cases in which in-hospital mortality would not be recorded are those where a patient is admitted more than 30 days after being given a diagnosis. However, even when also including 30-day post diagnosis outcomes, we still found no effect. The difference in mortality between the study by Hautz et al. and our study may therefore be due to other methodological differences such as size of study population, the single-centre nature of the study vs. national analysis, all ED diagnoses vs. selected ED diagnoses.

### Definition of diagnostic discrepancy

A change in diagnosis is not always due to error. For erysipelas, a patient may be admitted to the ED with sepsis, which happened in 4.80% of erysipelas DD episodes. When this life-threatening condition is under control, the ED staff could conclude that sepsis was related to erysipelas, therefore changing the diagnosis to erysipelas. The same situation can be found in DD of hip fracture episodes; a hip fracture diagnosis requires x-ray to confirm the diagnosis. It can be discussed whether, e.g., first assigning the diagnosis S70.0 Contusion of hip (8.23%) or S32.5 Fracture of pubis (1.86%) is a flaw or just the natural order in which patients awaiting diagnostic imaging are diagnosed. Furthermore, the admission diagnosis is also influenced by the inherent uncertainty characterising patients’ symptom reporting, which is evidently also affected by their physical and/or mental state at admission. For example, delirium or unconsciousness may radically change patient-physician communication. Delirium is a condition commonly related to, e.g., pneumonia or dehydration [37], which was recorded as admission diagnoses among both patient groups (J18.9 pneumonia: hip fracture 1.78%, erysipelas 2.09%, E86.9 dehydration: hip fracture 1.65%, erysipelas 2.09%). Alternatively, DD may also arise if the main complaint is trouble breathing (related to pneumonia), and the physician observes that the patient also suffers from erysipelas. Even though patients with DD might not be assigned to the DD category due to diagnostic errors made by the ED staff, the DD definition still captures some patient complexity that can be difficult for ED staff to handle and which requires their attention in order to improve patient outcomes.

### Possible explanation of study results

Emergency medicine has only recently (2017) been approved as a medical specialty in Denmark [38]. Hence, during the study period, few senior physicians with emergency medicine competencies were available at EDs, and staff had few incentives to work at the ED and stay in this medical field [12, 16, 39]. During the study period, the EDs were therefore highly dependent on senior physician resources outside the ED. Seniors employed at the ED were primarily recruited from other specialties than emergency medicine (some senior physicians in emergency medicine could even have been recruited from abroad). Senior physician employment was found to be associated with DD, which could indicate a mismatch of ED resources, where seniors were diagnosing patients harbouring diseases that did not belong to their medical specialty. This is supported by previous studies indicating that diagnostic error occurs when information-processing capacity (e.g. clinical experience from the ED) does not match information-processing demands (e.g. ED patients in need of a diagnosis and treatment) [1, 40] and that DD was often related to faulty information processing [5]. Thus, physicians will tend to look for information that confirms their intuition, and information that does not confirm this intuition will most often be rejected [1]. Physicians’ intuition is based on pattern recognition memorised through medical training. An orthopaedic surgeon would therefore be likely to find patterns of orthopaedic diagnoses, whereas an emergency medicine physician would be expected to have an eye for acute conditions. Another aspect of this problem is that physicians have been found to be poor at self-assessing their ability to diagnose patients. This tendency was most outspoken among physicians who were least experts [41], whereas physicians with higher expertise where more capable of distinguishing easily diagnosed cases from more complex ones. Hence a solution to this problem would be to let experts handle patients, in this case seniors with competencies matching patients’ needs. This was also indicated by the negative association between DD and availability of external senior physicians, since they were called upon only when a patient’s symptoms matched the medical specialty of the external senior physician. Hopefully, emergency medicine senior physicians would soon also fulfil this expert role at the ED.

### Limitations

In the field of diagnostic error, this study is unique owing to its long study period and the inclusion of episodes encountered at several EDs (national analyses). Another strength is the complete survey data providing information about the organisational determinants of this study. However, our survey data have some limitations: the long study period might increase the risk of recall bias, and high staff turnover in the study period is expected to decrease the precision of the timeline construction, since the respondent might not have been affiliated with the ED during the whole study period. From our survey, we know when the EDs started to employ senior physicians, but we do not know the number of employed senior physicians and if this changed over time. Another limitation of this study is that we do not have all clinical data and therefore cannot go into further detail and determine whether DD was related to diagnostic error. The lack of detail also means that we have limited possibility to adjust for episode complexity, e.g. in the form of triage scores, although we adjusted for comorbidity and age.

The definition of the study cost perspective (episode costs) is both a strength (focus on ED services) and a limitation (lack of measures capturing the societal effect of DD). As opposed to the diagnosis-related grouping (DRG) tariff (based on national averages), the data on which this outcomes measure is based provide the number of available tariffs and thereby the actual variation in episode costs, which is a major strength. Unfortunately, this database suffers from missing data. Our mixed effect models are capable of handling missing data [29, 30].

## Conclusion

Employing senior physicians at the ED would be expected to bring valuable resources to the ED, improving patient flow and improving diagnostic quality at the ED. However, this does not seem to be the case, maybe due to lack of appropriate emergency medicine competences at the ED. As indicated by our results, the consequences of DD are substantial. By considering the organisational determinants of DD, we are also in a position to suggest where our organisational efforts are most valuable. One could argue that we might already be moving in the right direction by increasing EM competencies at the ED (educating physicians). Further research is needed, covering an updated time period, to assess the long-term effects of this improvement in ED resources, and more patient groups must be added to the study population to improve the external validity of the study.

## Data Availability

Data that support the findings of this study are available from Statistics Denmark but restrictions apply to the availability of these data, which were used under license for the present study, and so are not publicly available.

## Abbreviations

DD: Diagnostic discrepancy
DRG: Diagnosis-related grouping
ED: Emergency department
EM: Emergency medicine
ICD-10: International Classification of Diseases version 10
